# A Case of Early-Stage Acute Myocarditis in a Child Detected by Focused Cardiac Ultrasound

**DOI:** 10.7759/cureus.100216

**Published:** 2025-12-27

**Authors:** Ci Zhuang Teh, Takaaki Mori

**Affiliations:** 1 Department of Emergency Medicine, Kandang Kerbau (KK) Women's and Children's Hospital, Singapore, SGP

**Keywords:** focused cardiac ultrasound, pediatric emergency department, point-of-care ultrasonography, viral-induced myocarditis, young child

## Abstract

Myocarditis in children is a life-threatening condition requiring prompt diagnosis and intervention. Its nonspecific signs and symptoms often mimic those of common viral illnesses and pose significant diagnostic challenges. Focused cardiac ultrasound (FoCUS) has recently been used for cardiac assessment in paediatric emergency care; however, reports on its use in diagnosing myocarditis are limited. We herein report a case of early-stage acute myocarditis detected by FoCUS. A previously healthy two-year-old girl presented with a three-day history of fever, and vomiting for one day. During the initial assessment, the patient was lethargic with slight tachycardia, but the physical examination results were unremarkable. She was treated for viral gastritis with oral ondansetron and a oral rehydration solution. However, she exhibited worsened tachycardia with a heart rate of 150 beats per minute and a gallop rhythm after the initial treatment. Electrocardiography showed sinus rhythm with a right bundle branch block; however, chest radiography was normal. FoCUS revealed a moderately reduced ejection fraction and distended inferior vena cava, suggesting acute myocarditis. She was referred to a paediatric cardiologist and echocardiography revealed similar findings. Cardiac enzymes levels were significantly elevated with a troponin T of 15,089 ng/l. Acute myocarditis was diagnosed, and the patient was admitted to the intensive care unit, where she underwent extracorporeal membrane oxygenation (ECMO). The patient was hospitalized for 13 days and was then discharged. FoCUS enables direct assessment of cardiac function, facilitating early diagnosis of critical conditions and improving outcomes.

## Introduction

Myocarditis is a potentially life-threatening condition caused mainly by infectious agents and is characterized by inflammation of cardiac myocytes, which may lead to myocardial edema, injury, or necrosis [1]. Although pediatric myocarditis is rare, its mortality remains high, ranging from 10% to 30% [1, 2]. Early recognition and treatment are essential to prevent severe complications [3].

Diagnosis in children is difficult because symptoms are often nonspecific and may resemble respiratory or gastrointestinal infections, particularly in infants. Only a minority of patients present with hepatomegaly or clear cardiac abnormalities [3-6]. Electrocardiography and cardiac biomarkers can support the diagnosis, but ECG findings vary widely and lack diagnostic accuracy, while delays in biomarker results limit their usefulness in the early phase [7]. These factors contribute to frequent delays in identifying acute myocarditis in children.

Point-of-care ultrasound (POCUS) has become increasingly used in pediatric emergency medicine because it is non-invasive and provides rapid bedside assessment [8]. Focused cardiac ultrasound (FoCUS), a targeted form of cardiac POCUS, has been introduced into pediatric emergency practice [9] and is commonly used to assess pericardial effusion and global ventricular function [10]. FoCUS is particularly valuable in suspected myocarditis because it allows immediate evaluation of systolic function and subtle ventricular changes at the bedside, even before laboratory or ECG findings become available. However, reports describing its use in diagnosing early-stage pediatric myocarditis remain limited.

Here, we describe a case in which FoCUS-enabled early detection of myocarditis, leading to timely diagnosis and intervention.

## Case presentation

A two-year-old girl presented to the emergency department with a three-day history of fever, cough, and rhinorrhoea, accompanied by seven episodes of vomiting and epigastric pain over the past day. She looks lethargic but her vital signs were unremarkable except for slight tachycardia: respiratory rate 30 breaths per minute, heart rate 132 beats per minute, blood pressure 87/67 mmHg, , SpO_2_ 100% on room air, temperature 38.2℃. Physical examination revealed that her lungs were clear without wheezing, and her heart did not show any murmurs or gallop rhythm. The abdomen was soft and did not exhibit abdominal tenderness or organomegaly. She had good perfusion and warm peripheries with a capillary refilling time (CRT) of less than two seconds. Viral gastritis was initially suspected, and oral ondansetron, paracetamol, and rehydration solutions were administered. During reassessment one hour after the oral rehydration therapy, the patient exhibited worsening tachycardia with a heart rate of 160 beats per minute, despite resolution of fever with a temperature of 37.0℃. Blood pressure was 102/52 mmHg, respiratory rate was 36 breaths per minute, and SpO_2_ 100% on room air. Repeated physical examinations revealed a gallop rhythm on auscultation. All other systemic findings were unremarkable.

Due to the worsening tachycardia and gallop rhythm, a paediatric emergency physician with more than five years of experience in paediatric POCUS performed FoCUS using a SonoSite EDGE Ⅱ (FUJIFILM SonoSite, Inc., Bothell, WA, USA) equipped with a phased array transducer (4-8 MHz). The patient was placed in the supine position, and the transducer was placed over the left fourth intercostal space with the probe indicator towards the patient’s right shoulder (PLAX: parasternal long axis view), then the left patient’s shoulder (PSAX: parasternal short axis view). Subsequently, the transducer was placed beneath the xiphoid process with the probe indicator directed towards the patient’s head (subxiphoid view). PLAX and PSAX views revealed global hypokinesis with a significantly decreased visual ejection fraction (EF), but no obvious pericardial effusion or structural abnormalities were found (Videos 1, 2).

**Video 1:** FoCUS (parasternal long axis view: PLAX) FoCUS showed significant decreased left ventricular contractility with decreased visual left ventricular ejection fraction (<50%), but did not show any anechoic structure suggesting pericardiac effusion around the left ventricle. FoCUS: Focused cardiac ultrasound.

**Video 2:** FoCUS (parasternal short axis view: PSAX) FoCUS showed significant decreased left ventricular contractility with decreased visual left ventricular ejection fraction (<50%), but did not show any anechoic structure suggesting pericardiac effusion around the left ventricle. FoCUS: Focused cardiac ultrasound.

The subxiphoid view also showed a distended inferior vena cava (IVC) (Video 3).

**Video 3:** FoCUS (subxiphoid inferior vena cava view) FoCUS showed distended inferior vena cava without respiratory variation. FoCUS: Focused cardiac ultrasound.

**Figure 1:**
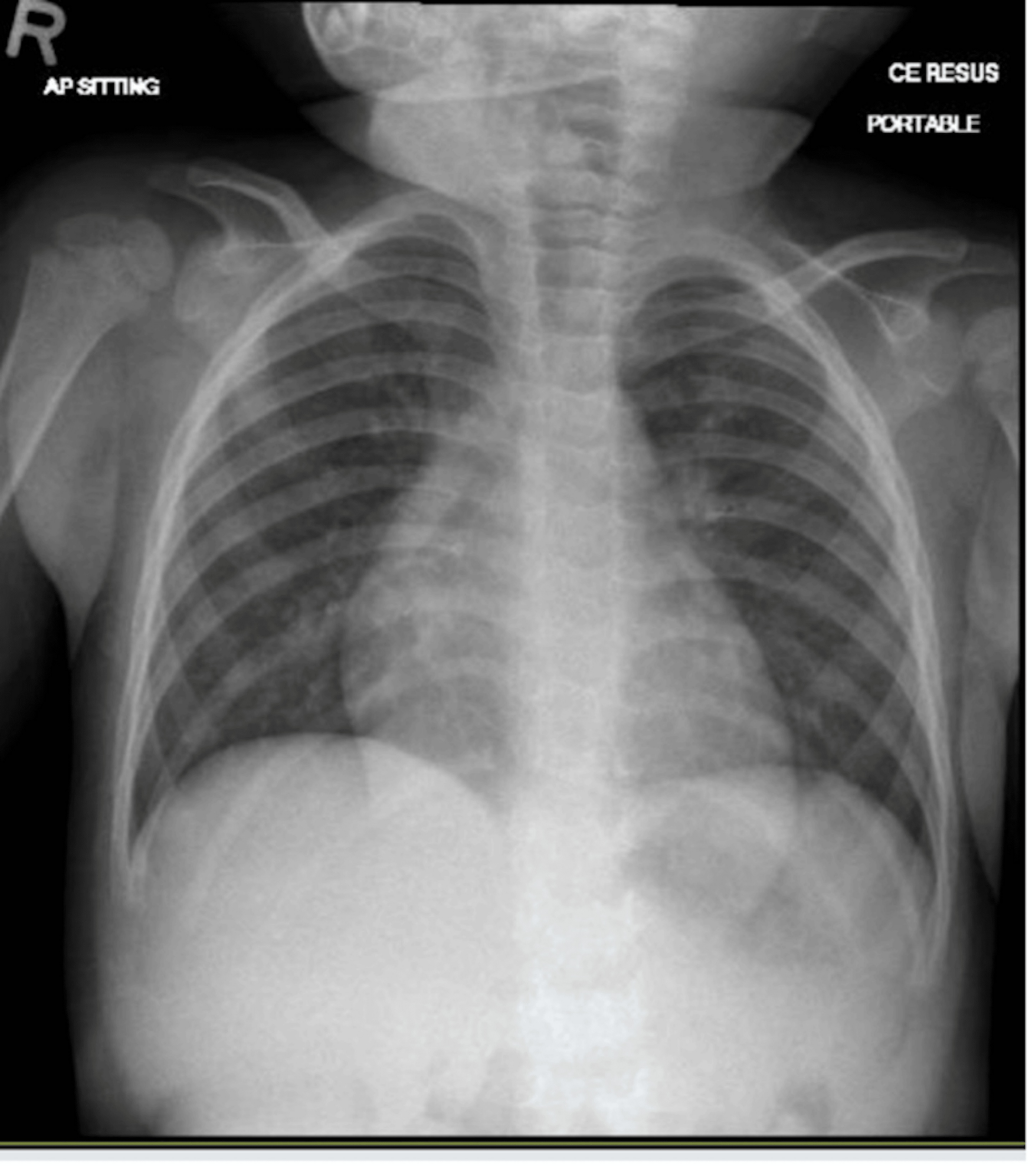
Chest radiograph Chest radiograph does not show cardiomegaly nor pulmonary oedema

Chest radiographs did not show cardiomegaly (Figure 1).

**Figure 2:**
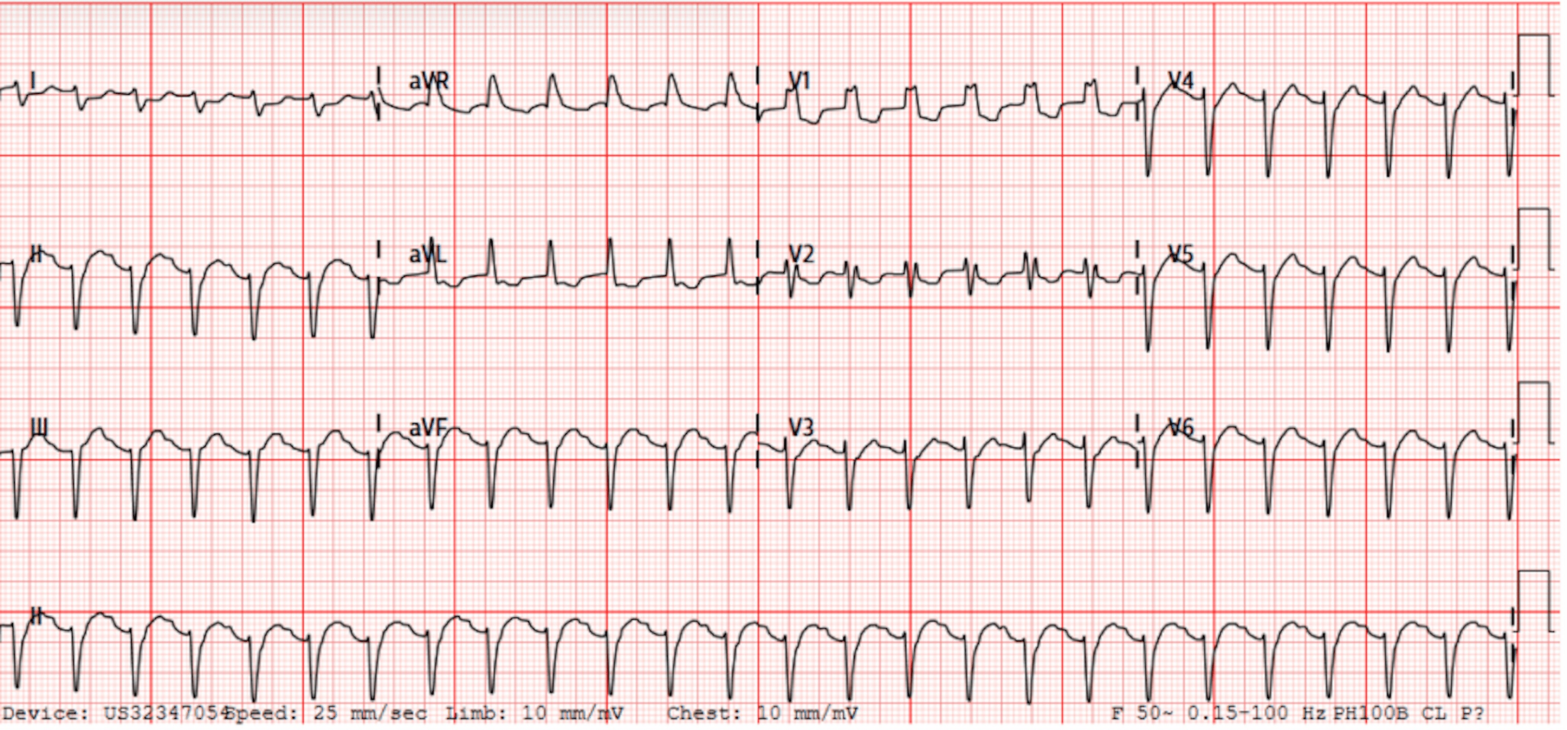
Electrocardiogram Electrocardiogram showed normal sinus rhythm with right bundle branch block.

ECG showed sinus rhythm with right bundle block but no ST-T changes (Figure 2).

**Figure 3:**
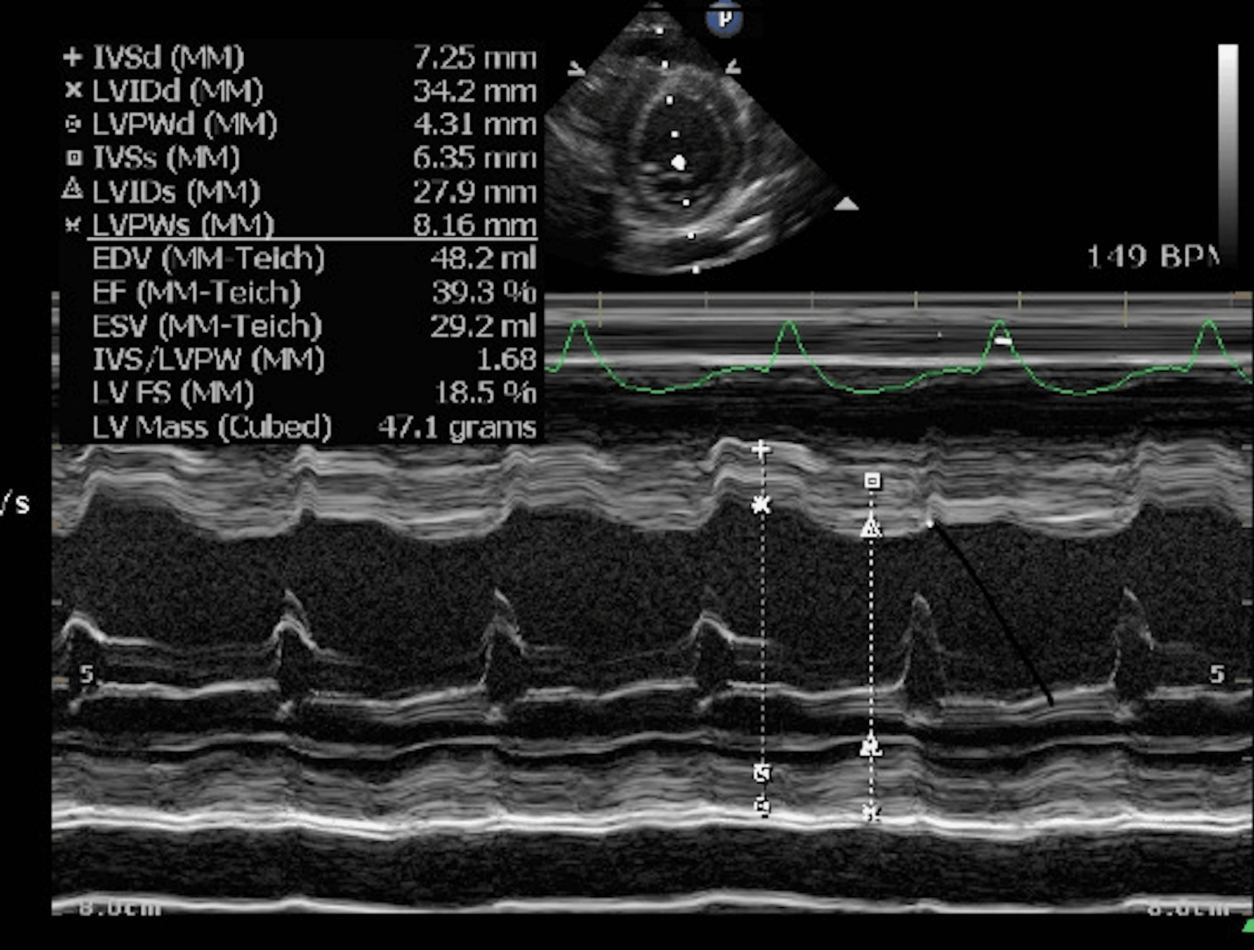
A cardiologist-performed echocardiogram A cardiologist-performed echocardiogram demonstrated a moderately reduced left ventricular systolic function with an ejection fraction (EF) of 39%.

**Figure 4:**
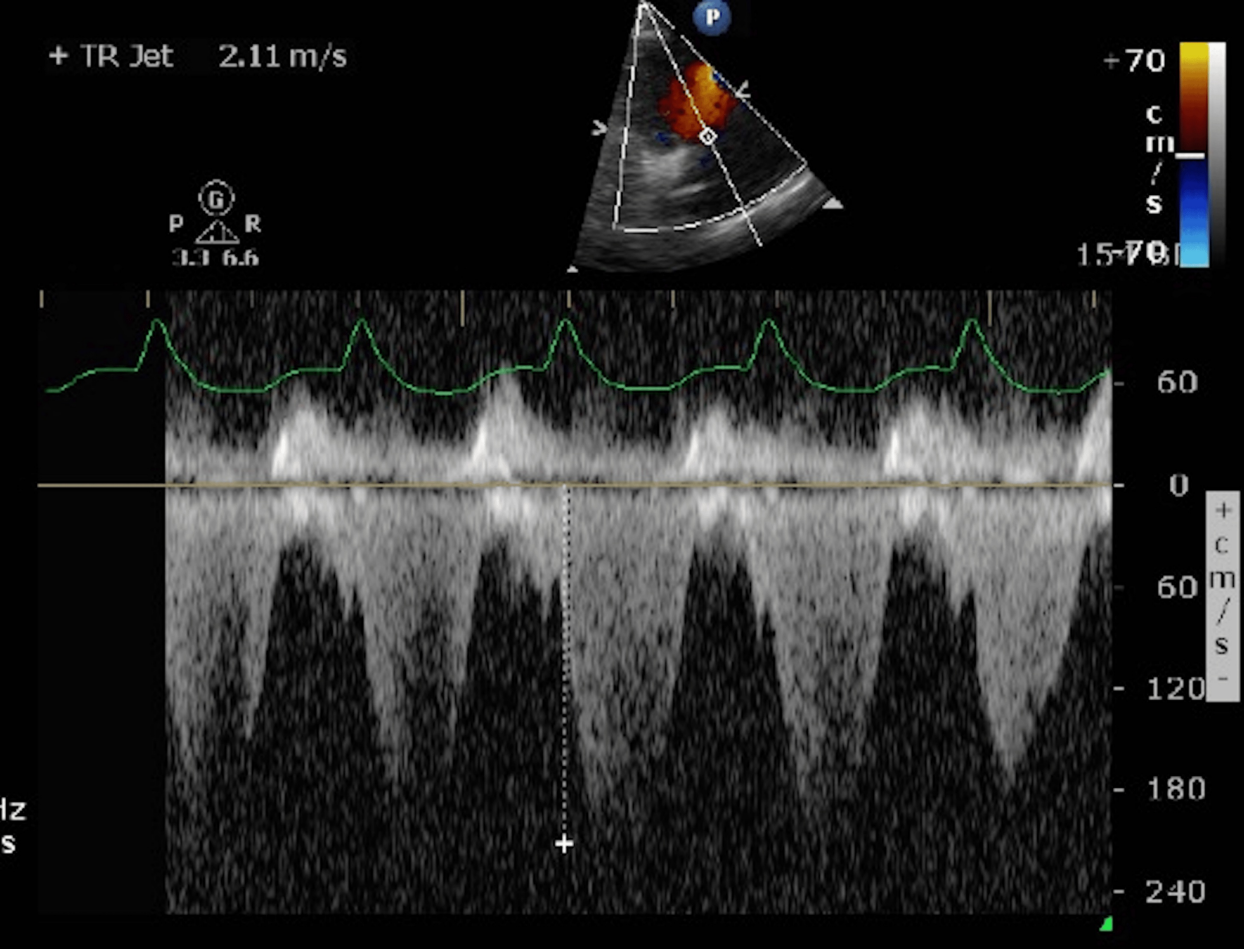
A cardiologist-performed echocardiogram A cardiologist-performed echocardiogram demonstrated mild tricuspid regurgitation.

**Figure 5:**
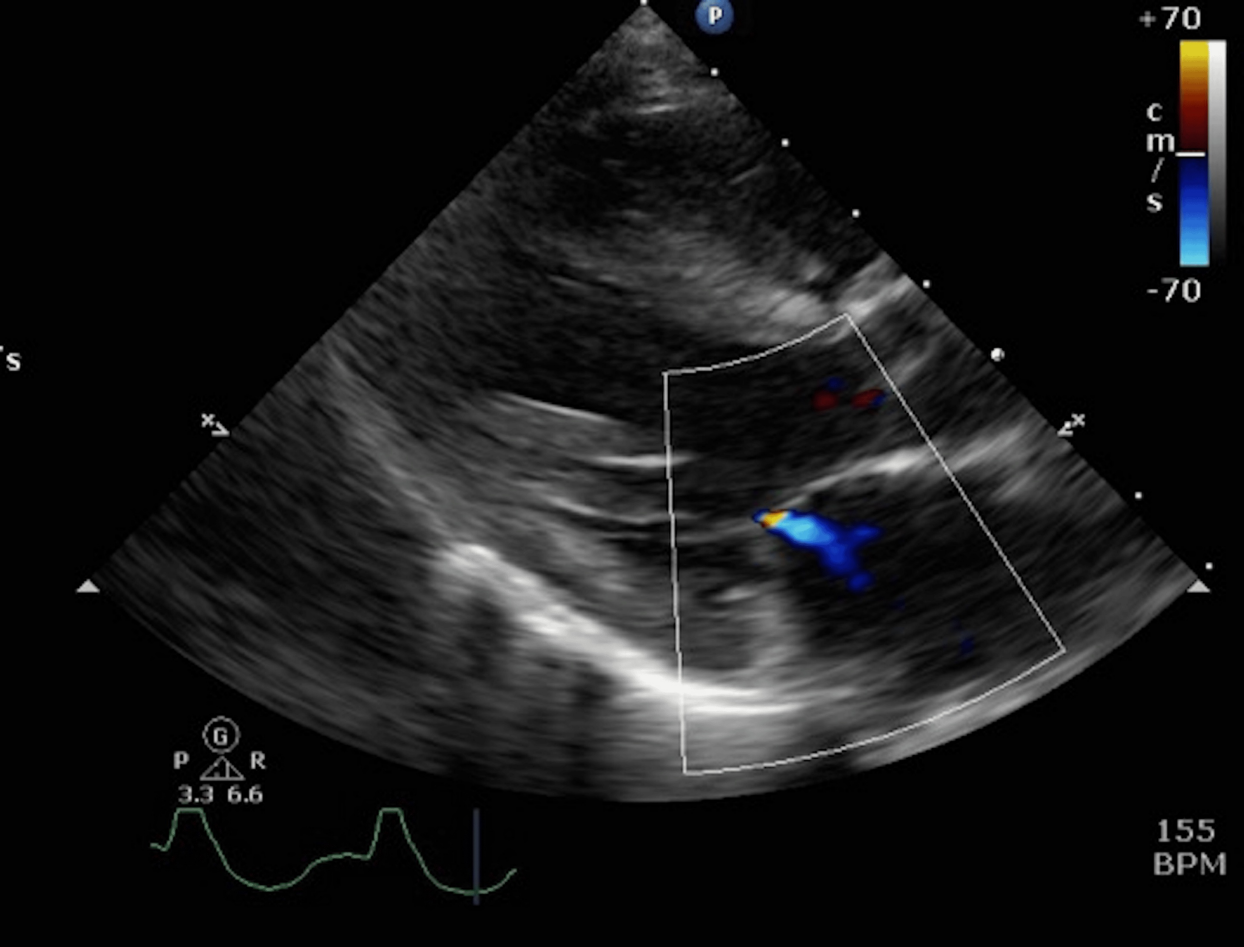
A cardiologist-performed echocardiogram A cardiologist-performed echocardiogram demonstrated mild mitral regurgitation.

Based on physical examination and FoCUS findings, myocarditis with compensated cardiogenic shock was suspected. The patient was promptly referred to a cardiologist and subsequently transferred to the cardiac intensive care unit where continuous intravenous adrenaline (0.05 microgram/kg/min) was administered. A cardiologist-performed echocardiography demonstrated a moderately reduced left ventricular systolic function with an EF of 39%, along with mild mitral and tricuspid regurgitation (Figures 3-5).

These findings were consistent with the initial FoCUS assessments in the emergency department. Blood tests revealed significant derangements, including elevated cardiac enzyme levels (Table 1).

**Table 1:** Laboratory test results NT-proBNP: N-terminal pro-B-type natriuretic peptide; MB: myocardial band (in creatine kinase-MB).

Blood test		Normal range
White Blood Cell	16.77x10^9 ^L	5.22-13.35x10^9 ^L
Haemoglobin	12.6 g/dL	11.4-14.2 g/dL
Platelet	454 x 10^3 ^L	140-440 g/dL
Sodium	135 mmol/L	138-145 mmol/L
Potassium	5.4 mmol/L	3.4-4.7 mmol/L
Chloride	110 mmol/L	98-107 mmol/L
Bicarbonate	10 mmol/L	14-22 mmol/L
Urea	8.9 mmol/L	3.2-7.9 mmol/L
Creatinine	44 mmol/L	18-38 mmol/L
Troponin I	15089 ng/L	≦10 ng/L
Creatine Kinase	860 U/L	NA U/L
Creatine Kinase-MB	31.2 IU/L	<65 IU/L
NT-proBNP	>70000 pg/mL	NA pg/mL

A nasopharyngeal viral swab revealed positive results for adenovirus, parvovirus, and rhino/entero viruses.

Acute myocarditis was diagnosed, and extracorporeal membrane oxygenation (ECMO) was planned. During ECMO initiation, the patient experienced loss of cardiac output, requiring 30 seconds of cardiopulmonary resuscitation (CPR) before the return of spontaneous circulation. ECMO was successfully initiated and maintained for four days, followed by successful decannulation. After ECMO termination, intravenous milrinone was administered for another three days. The patient was hospitalized for 13 days and discharged on oral captopril at 3 mg/kg/day.

## Discussion

Myocarditis is an inflammatory condition of the heart, triggered by various infectious and non-infectious factors [11]. It not only carries a high mortality rate but also can result in long-term complications, including dilated cardiomyopathy, chronic heart failure, and sudden cardiac death (SCD), particularly in young adults [12]. A retrospective study reported the overall incidence rate of myocarditis as 1.95 per 100,000 person-years. However, because some patients remain asymptomatic, the true incidence of myocarditis remains unknown [13].

Diagnosing myocarditis is often challenging owing to its nonspecific and variable presentation. Symptoms often overlap with more common conditions such as viral infections and fever, which occur in around 50% of cases [3-6]. Other symptoms include upper respiratory tract infections (32%) and gastrointestinal disturbances (6%), which may lead to missed diagnoses, especially in the absence of classic signs such as gallop rhythm or organomegaly [3-6]. Clinicians must maintain a high index of suspicion, particularly in patients who fail to respond to initial treatment. In the present case, the patient presented with epigastric pain and vomiting mimicking viral gastritis. 

Chest radiographs, ECG, and cardiac biomarkers are frequently used to guide the diagnosis of myocarditis but often yield nonspecific findings. Chest radiographic features may include pulmonary edema and cardiomegaly; however, their sensitivity is modest (55%), so normal chest radiograph does not exclude myocarditis [5]. ECG findings may range from normal to nonspecific ST-T wave changes or show ventricular/atrial arrhythmias or high-grade atrioventricular block in up to 45% of pediatric cases of myocarditis [3,5,14] despite a sensitivity of 93% [5]. A study identified a cardiac troponin T cutoff of 0.052 ng/ml for diagnosing acute myocarditis, with 71% sensitivity and 86% specificity [15]. However, the turnaround time for results limits its value in the immediate diagnosis of myocarditis. In the present case, chest radiography and ECG did not reveal any significant abnormalities, possibly due to the early stages of acute myocarditis. Troponin T levels were significantly elevated, but the result was revealed after the patient transfer to the cardiac intensive care unit (CICU).

Although cardiac magnetic resonance imaging is considered as the gold standard, its use in the acute settings is often limited by logistical constraints and the requirement for patient stability. Endomyocardial biopsy remains the definitive diagnostic modality, providing valuable histological insights; however, it is invasive and not feasible for routine emergency evaluation [16]. Given these limitations, there is increasing emphasis on alternative diagnostic approaches that enable rapid bedside assessments.

FoCUS has emerged as a valuable adjunct, offering a non-invasive, readily accessible bedside tool for cardiac assessment. It enables targeted evaluation of the heart using specific views, including the PLAX, PSAX, apical four chamber (Ap4), and subxiphoid IVC views. FoCUS facilitates real-time evaluation of left ventricular EF, regional wall motion abnormalities, pericardial effusion, ventricular dilation, hypertrophy, and diastolic dysfunction [17]. A study demonstrated that the kappa statistics of agreement between pediatric emergency physicians performed FoCUS and cardiologist-performed echocardiography for detection of left ventricular function and IVC collapsibility were high at 0.87 and 0.73, respectively [18]. Another study found that pediatric intensivist-performed FoCUS showed 100% sensitivity and greater than 85% specificity for identifying left ventricular dysfunction compared with cardiologist-performed echocardiography [19]. These findings indicate that FoCUS serves as a valuable tool for real-time cardiac assessment in pediatric emergency care although its accuracy remains dependent on image acquisition and interpretation skills.

In pediatric emergency and critical care settings, FoCUS has been used for rapid cardiac assessment in various clinical scenarios, including cardiac arrest, systolic or diastolic heart failure, pericardial effusion, pulmonary embolism, aortic dissection, and hypertrophic obstructive cardiomyopathy [9]. However, the reports on its use in diagnosing acute myocarditis are lacking. A case report showed that FoCUS revealed a slight pericardial effusion and significantly decreased left ventricular EF aiding in the diagnosis of acute myocarditis in a five-year-old girl presenting with viral infection-like symptoms [20]. However, the patient exhibited signs of shock including tachycardia and weak peripheral pulses with prolonged CRT. Chest radiography and ECG at the time of diagnosis showed pulmonary edema and ST-T elevation, respectively. Another case report also showed that FoCUS revealed a dilated left ventricle and decreased EF, suggesting acute myocarditis, although the case was a referral in which abnormal ECG and troponin T findings had already been found, and the patient had a systolic murmur in the heart and bilateral crackles in her lungs [21]. In the present case, FoCUS played a significant role in diagnosing the early stage of acute myocarditis as the patient exhibited only tachycardia and gallop rhythm on auscultation, without any abnormalities in the chest radiograph and ECG findings in contrast to aforementioned case reports (Table 2).

**Table 2:** Comparison between previous reports and the present case CRT: Capillary refill time; IVC: Inferior vena cava; LVEF: left ventricular ejection fraction; aVL: augmented voltage left arm; RBBB: right bundle brunch block

Authors	Age (years old)	Vital signs (abnormal)	Physical examinations	Chest X-ray	ECG	Laboratory data	FoCUS	Echocardiography
	Sex							
Gupta A et al. [20]	5	Tachycardia (159/bpm), tachypnea (66/minutes)	Prolonged CRT (3-4 seconds)	Pulmonary edema	ST-T elevation in Ⅰ, aVL, V5-6	Elevated troponin level	Decreased LVEF, distended IVC	n.a. (cardiology agreed the diagnosis)
	Female							
Poteh NA et al. [21]	14	Slight tachycardia (98/bpm)	Systolic murmur, faint cracle	n.a.	n.a.	Elevated troponin level	Decreased LVEF	Decreased LVEF, mild dilatation of LV and ectasia of the coronary arteries
	Male							
Present case	2	Worsened tachycardia (160/bpm)	Gallop rhythm	Normal	RBBB	Elevated troponin level	Decreased LVEF, distended IVC	Decreased LVEF, distended IVC, mild mitral and tricuspid regurgitation
	Female							

While no studies have assessed the diagnostic accuracy of FoCUS in identifying acute myocarditis in children, it should be considered when evaluating clinically unhealthy patients, as it can aid in the early detection of myocarditis.

Thus, this case report adds to the growing body of evidence suggesting that FoCUS, when performed by a paediatric emergency physician, is a valuable tool for diagnosing acute myocarditis in children. FoCUS lacks objective measurements, and the limited view or imaging window sometimes affects the accuracy of the analysis, and cannot replace a comprehensive cardiac echocardiogram [17]. In our institution, the FoCUS operators had previously been instructed on FoCUS in formal ultrasound training courses, received at least a one-hour lecture and training session in the pediatric emergency department (PED), or had experience of education on echocardiography in their pediatric cardiology training. Although minimum requirements for FoCUS operators have not yet been established, POCUS experts at our institution supervise or review FoCUS studies performed in our pediatric emergency medicine (PEM) setting. To ensure the quality of FoCUS examinations, minimum competency standards should be defined in line with the previously published consensus statement [22]. Furthermore, image acquisition and interpretation require training and experience due to operator dependence. However, paediatric emergency physicians can be equipped with this useful modality to enhance their diagnostic capabilities.

## Conclusions

Myocarditis in children is a life-threatening condition, yet its diagnosis remains challenging due to nonspecific symptoms, and subtle physical examination findings. FoCUS provides real-time assessment of cardiac contractility, allowing for early detection of myocardial dysfunction. FoCUS can play a crucial role in the early diagnosis of acute myocarditis in paediatric emergency care, although it remains an adjunct diagnostic tool.
